# Supplementary material for: N-Way NIR Data Treatment through PARAFAC in the Evaluation of Protective Effect of Antioxidants in Soybean Oil
Source: Molecules. 2020 Sep 23;25(19):4366. doi: 10.3390/molecules25194366 (PMC7583810; doi:10.3390/molecules25194366)
Supplement: Supplementary file 1 [file molecules-25-04366-s001.pdf]

Supplementary Materials

Table S1. UV absorption of oil samples heated at 150° C.

Sample	230 nm	270 nm
**C**	1.7459	1.8089
**BHT**	2.0039	2.0141
**TBHQ**	1.9409	2.0051
**PHE**	1.9678	2.2410
**DGBE**	1.6831	1.7779
**PPSE**	1.7996	1.8534
**PSE**	1.8556	1.9036
**PH**	2.0209	2.1151
**DGB**	2.1511	2.1726
**PPS**	2.0843	2.1046
**PS**	2.1479	2.1779

C=Control. BHT= Butylated hydroxytoluene. TBHQ=Tert-butyl hydroquinone. PHE=Provençal herbs extract. DGBE=Dehydrated goji berry extract. PPSE=Pumpkins seed extract. PSE=Poppy seed extract. PH=Provençal herbs. DGB=Dehydrated goji berry. PPS=Pumpkins seed. PS=Poppy seed.

.
